# Lignin- and Silver-Modified Multifunctional Cotton Fabrics: Influence of α- and β-Chitosan Pretreatment on Structure–Property Relationships

**DOI:** 10.3390/polym18111279

**Published:** 2026-05-22

**Authors:** Sirachat Nongsok, Chutima Vanichvattanadecha, Penwisa Pisitsak

**Affiliations:** 1 Department of Materials and Textile Technology, Faculty of Science and Technology, Thammasat University, Pathum Thani 12121, Thailand; sirachat.nongsok@gmail.com; 2 Advanced Composite and Nanotextiles Research Team, National Nanotechnology Center, National Science and Technology Development Agency, Pathum Thani 12120, Thailand; 3 Center of Excellence on Petrochemical and Materials Technology, Chulalongkorn University, Bangkok 10330, Thailand

**Keywords:** lignin, chitosan polymorph, silver nanoparticle, cotton, antibacterial activity, UV protection

## Abstract

This study investigates lignin as a renewable functional dye capable of simultaneously imparting coloration and multifunctional performance to cotton textiles, with particular emphasis on how chitosan polymorphs influence lignin-mediated silver nanoparticle (AgNP) systems. Cotton fabrics were pretreated with α- or β-chitosan crosslinked with glyoxal and subsequently dyed with lignin in the presence of silver ions to generate lignin-mediated AgNPs. Inductively coupled plasma optical emission spectrometry (ICP–OES) analysis showed that α-chitosan retained a higher silver content (40.7 mg/kg) than β-chitosan (14.7 mg/kg). Transmission electron microscopy (TEM) revealed that α-chitosan produced larger AgNPs (≈13.6 nm), whereas β-chitosan was associated with smaller measurable nanoparticles (≈4.3 nm). Despite lower silver loading, β-chitosan–modified fabrics exhibited higher antibacterial activity against *Staphylococcus aureus* (82.6%) than α-chitosan-modified fabrics (68.7%). These results suggest that antibacterial performance in lignin–silver coating systems may depend not only on silver loading, but also on the distribution and accessibility of active components within the coating layer. In addition, the coatings improved UV protection, tensile properties, and color strength. Overall, the findings demonstrate that chitosan polymorphism plays an important role in controlling nanoparticle characteristics and multifunctional performance in lignin-based textile systems.

## 1. Introduction

**Figure 1:**
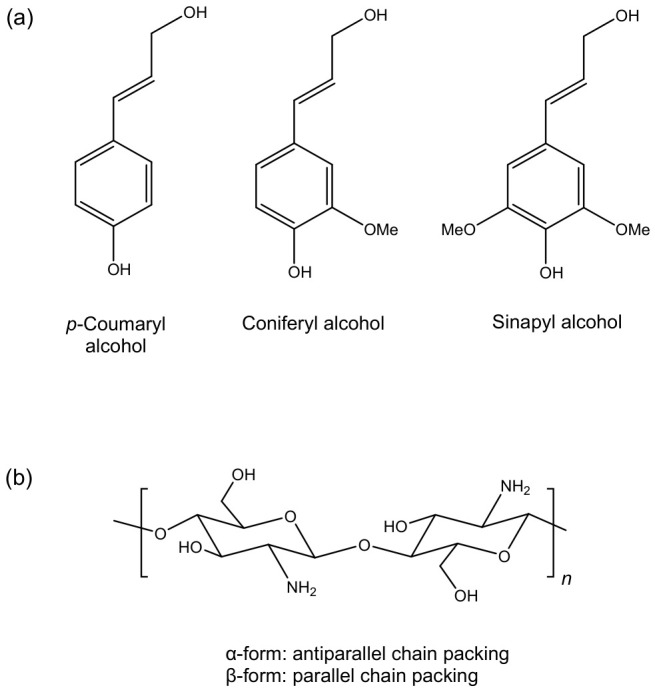
(**a**) Representative lignin monolignols; (**b**) repeating structure of chitosan and description of α- and β-form chain arrangements.

The growing demand for sustainable and multifunctional textiles has driven increasing interest in renewable biopolymers as alternatives to conventional petroleum-based finishing agents. Among these, lignin has attracted considerable attention as the second most abundant natural aromatic biopolymer, derived from wood and generated as a major byproduct of the paper industry [1]. Lignin, one of the most abundant natural aromatic biopolymers, is primarily composed of *p*-hydroxyphenyl (H), guaiacyl (G), and syringyl (S) units derived from *p*-coumaryl alcohol, coniferyl alcohol, and sinapyl alcohol, respectively (see Figure 1a) [2,3]. Commercial alkali lignin, commonly associated with kraft-type industrial processing, is generally considered to be guaiacyl-dominant, particularly for softwood-derived lignin. Due to its aromatic and phenolic functionalities, lignin can serve as both a natural dye and a functional additive, providing UV shielding, antioxidant, flame retardant, and antimicrobial properties [4,5,6,7]. In addition, phenolic and aliphatic hydroxyl groups in lignin can participate in the reduction of Ag^+^ ions, facilitating the formation of silver nanoparticles (AgNPs) [8]. Previous studies have demonstrated that lignin can function as both a reducing and stabilizing agent for AgNP synthesis [5,9]. For example, Aadil et al. reported the green synthesis of lignin-mediated AgNPs exhibiting effective antibacterial activity [10]. Similarly, Jaiswal et al. developed lignin-mediated AgNP hydrogels for wound dressing applications and demonstrated strong antibacterial activity against both *S. aureus* and *E. coli* [11]. The antibacterial activity of silver has been associated with both silver ions (Ag^+^) and metallic AgNPs. AgNPs may exhibit enhanced antibacterial performance due to their high surface-to-volume ratio, which increases interactions with bacterial cell membranes and facilitates the release of bioactive silver species. In addition, nanoparticle size, aggregation state, and surface accessibility have been reported to strongly influence antibacterial efficiency [12]. In addition, synergistic effects between lignin and AgNPs have been reported, where the combination enhanced antibacterial and UV-protective performance compared with individual components alone [13]. Such lignin-mediated nanoparticle systems may therefore be promising for textile finishing applications requiring antibacterial and UV-protective performance.

Chitosan, another bio-based material, has been widely studied for sustainable textile finishing due to its film-forming ability, cationic nature, and strong affinity for negatively charged species [14,15]. The presence of protonated amino groups enables chitosan to effectively interact with anionic compounds, thereby enhancing their adsorption and fixation on textile substrates. In addition, chitosan exhibits a strong affinity toward cellulose fibers through hydrogen bonding between the hydroxyl groups of cellulose and the amino groups of chitosan, together with film formation on fiber surfaces. This surface modification significantly improves the dyeability of cotton with natural dyes, which are typically difficult to fix because cellulose surfaces generally carry negative charges under neutral to alkaline conditions. Under acidic conditions (pH < 6.5), protonation of amino groups imparts positive charges to chitosan, promoting electrostatic interactions with anionic species [14,16,17,18]. These characteristics make chitosan suitable as a surface-binding matrix for lignin-based textile systems, where protonated amino groups may promote interactions with lignin species and improve their incorporation onto cotton fibers.

Chitin exists in three polymorphic forms—α, β, and γ—based on the arrangement of carbohydrate chains. The corresponding chitosan inherits these structural characteristics; however, the α- and β-forms are the most prominent and widely studied due to their availability and practical relevance (see Figure 1b). The α-form, commonly derived from crustacean shells, consists of an antiparallel chain arrangement stabilized by strong hydrogen bonding, resulting in high structural stability. In contrast, β-chitosan, typically derived from β-chitin in squid pen, exhibits a parallel chain arrangement with weaker intermolecular interactions, leading to lower crystallinity, a more open structure, and higher reactivity than the α-form [19,20]. These structural differences are expected to influence not only the uptake of lignin and metal species but also their distribution and accessibility within the coating layer.

Although previous studies have compared α- and β-chitosan [20], and chitosan/lignin multilayer coatings with immobilized AgNPs have been reported to impart antibacterial and UV-protective properties to cotton textiles [21], the influence of chitosan polymorphism on lignin-mediated AgNP systems remains largely unexplored. α- and β-chitosan exhibit distinct chain packing arrangements and intermolecular interactions, which may influence the incorporation, accessibility, and distribution of lignin and silver species within the coating layer. In addition, Ravishankar et al. reported that alkali lignin could diffuse into chitosan matrices and reduce intermolecular interactions within chitosan, leading to a more amorphous and open structure suitable for functional biomaterial applications [22].

Based on these considerations, this study aims to develop multifunctional cotton fabrics using lignin-mediated AgNP systems in the presence of α- or β-chitosan. We hypothesized that the distinct structural organization of α- and β-chitosan would influence the incorporation, distribution, and accessibility of lignin-mediated AgNPs within the coating layer. Cotton fabrics were first modified with glyoxal-crosslinked chitosan, followed by lignin dyeing in the presence of silver ions to generate lignin-mediated AgNPs. The effects of chitosan polymorphism on silver loading, nanoparticle characteristics, antibacterial activity against *Staphylococcus aureus*, UV protection, tensile properties, and color performance were systematically investigated.

## 2. Materials and Methods

### 2.1. Materials

A plain-woven 100% cotton fabric was used as the substrate (102.0 g/m^2^), with a yarn density of 69 ends per inch (27 ends cm^−1^) and 89 picks per inch (35 picks cm^−1^). Alkali lignin (average molecular weight Mw ~10,000, sulfur content approximately 4%, pH 10.5), a commercial industrial lignin associated with kraft-type processing, was purchased from Sigma-Aldrich (St. Louis, MO, USA) (Figure 1a). Silver nitrate (AgNO_3_, 99.8% purity) was obtained from Sigma-Aldrich (St. Louis, MO, USA). Glyoxal solution (40 wt.% in water, C_2_H_2_O_2_) was supplied by Acros Organics (Geel, Belgium). Citric acid (C_6_H_8_O_7_) was purchased from AJAX FineChem (Seven Hills, NSW, Australia). Alpha chitosan (α-chitosan, low molecular weight, Mw 50,000–190,000 Da) was obtained from Sigma-Aldrich (St. Louis, MO, USA), while beta chitosan (β-chitosan), extracted from squid pen with a degree of deacetylation of approximately 95% and a molecular weight of ~1,000,000 Da, was supplied by Bonafides Marketing Co., Ltd. (Bangkok, Thailand) (Figure 1b). Deionized (DI) water was used throughout all experiments.

### 2.2. Pretreatment of Cotton Fabrics with α- and β-Chitosan

Cotton specimens were cut into A4-sized sheets (approximately 8 g per specimen). The fabric was cleaned using a standard detergent solution (2.0 g/L) at a liquor ratio (liquid volume to fabric weight ratio, L:R) of 30:1. The washed fabrics were subsequently treated in 1.0 M sodium hydroxide solution at 90.0 °C for 1 h at L:R = 30:1. After treatment, the samples were rinsed repeatedly with DI water until neutral pH was reached, followed by air drying at room temperature.

Pretreatment solutions containing 1.5% *w*/*v* α-chitosan or 1.5% *w*/*v* β-chitosan and 2.0% *w*/*v* citric acid in aqueous solution were separately prepared. The pre-cleaned cotton fabrics were immersed in the pretreatment solutions at room temperature for 18 h under static conditions at L:R = 30:1. After impregnation, the fabrics were passed through a two-roll laboratory padding mangle (UT-TX393, LABTEC, Labtech Engineering Co., Ltd., Samut Prakan, Thailand) at a nip pressure of 1.0 kg/cm^2^. The padded samples were immediately weighed to determine wet pick-up, which was controlled to approximately 90–100% based on the weight difference before and after padding. Subsequently, the padded fabrics were immersed in an aqueous glyoxal solution (~4.0% *w*/*v*) at L:R = 30:1 and incubated at 40.0 °C for 1 h to promote crosslinking. After crosslinking, the fabrics were rinsed with DI water until a neutral pH was reached and then dried in an oven at 60.0 °C to a constant weight.

### 2.3. Preparation of Lignin–AgNPs and Dyeing Process

Lignin dye solutions were prepared at a concentration of 40.0% owf, corresponding to 0.8 g of lignin dissolved in 60.0 mL of deionized (DI) water (1.33% *w*/*v*). Cotton fabrics (approximately 2 g per sample) were dyed at L:R = 30:1.

For silver-containing dye baths, 2.0 mL of a 60.0 mM AgNO_3_ solution was added dropwise to 58.0 mL of the lignin solution under continuous stirring to obtain a final volume of 60.0 mL. This resulted in an AgNO_3_ concentration of 0.034% *w*/*v* (equivalent to 216 ppm Ag^+^), while maintaining the lignin concentration at 1.33% *w*/*v*.

For dyeing without silver, 2.0 mL of DI water was added to the lignin solution to achieve the same final volume, ensuring identical lignin concentration across all samples.

The pH of the dye bath was adjusted to 4.0 using 1.0 M citric acid added dropwise under continuous stirring. Upon the addition of AgNO_3_ and subsequent pH adjustment, the dye bath gradually changed to a darker brown color, suggesting the formation of lignin-mediated AgNPs. The reduction process is attributed to the reducing ability of lignin, while elevated dyeing temperature may further promote Ag^+^ reduction during the dyeing process. Dyeing was carried out using an exhaustion method in a laboratory-scale infrared (IR) dyeing machine (DAELIM STARLET, StarLet-3, Siheung-si, Republic of Korea). The process was conducted at 60.0 °C for 20 min, followed by heating to 90.0 °C and holding for 40 min. After dyeing, the bath was cooled to room temperature, and the samples were removed, rinsed thoroughly with DI water, and air-dried under ambient conditions.

To remove unfixed dye, the dyed fabrics were subjected to a soaping treatment using a standard detergent solution (2.0 g/L) at L:R = 30:1 in the IR dyeing machine at 60.0 °C for 20 min. The samples were then rinsed with DI water and air-dried at room temperature.

For clarity, the sample nomenclature used in this study is defined as follows: “Cot” denotes cotton fabric, “αCS” and “βCS” represent α- and β-chitosan pretreatments, respectively, “L” indicates lignin, and “Ag” refers to silver. For example, Cot-αCS-L corresponds to cotton fabric treated with α-chitosan and lignin without silver, whereas Cot-βCS-L-Ag refers to cotton fabric treated with β-chitosan, lignin, and silver.

### 2.4. Characterization

#### 2.4.1. Scanning Electron Microscopy and Energy-Dispersive X-Ray Spectroscopy (SEM–EDS)

The surface morphology and elemental composition of cotton fabrics dyed with silver-containing lignin were further analyzed using scanning electron microscopy (SEM) equipped with an energy-dispersive X-ray spectroscopy (EDS) system (SU15000, Hitachi High-Technologies Corporation, Tokyo, Japan). SEM was operated at 10.0 kV. Samples were sputter-coated with gold at 15.0 mA for 60.0 s before observation. SEM images were used to evaluate surface coverage and particle distribution, while EDS analysis was employed to confirm the presence and distribution of silver on the fabric surface.

#### 2.4.2. Determination of Total Silver Content in Treated Cotton Fabrics

Silver content was quantified using inductively coupled plasma optical emission spectroscopy (ICP-OES, Varian 730-ES, Varian Inc., Palo Alto, CA, USA). The samples were digested with nitric acid (65%) using microwave digestion according to EPA Method 3052 prior to analysis. The emission wavelength for silver (Ag) was set at 328.68 nm. After digestion, the solutions were diluted to an appropriate volume with deionized (DI) water and analyzed using external calibration with standard Ag solutions.

#### 2.4.3. Transmission Electron Microscopy (TEM)

The morphology of AgNPs formed within the cotton fabrics dyed with silver-containing lignin was investigated using transmission electron microscopy (JEM-2100 Plus, JEOL Ltd., Tokyo, Japan). AgNPs were extracted from the dyed fabrics by ultrasonic treatment (Soltec, Sonica 3200 S3, Milano, Italy) in DI water at room temperature for 4.5 h. The resulting suspension was filtered through Whatman No. 1 filter paper prior to TEM analysis. Particle sizes were measured from TEM micrographs using ImageJ software 1.54p (National Institutes of Health, Bethesda, MD, USA). Only clearly distinguishable and non-overlapping particles were included in the analysis, while severely agglomerated particles were excluded from size measurements. A total of 237 particles were measured for Cot-αCS-L-Ag, whereas only 30 particles could be measured for Cot-βCS-L-Ag due to the substantially lower abundance of detectable nanoparticles in the extracted dispersion.

#### 2.4.4. ATR-FTIR Analysis

Functional groups present on untreated cotton, surface-modified cotton, and lignin-dyed cotton fabrics were analyzed using attenuated total reflection Fourier transform infrared spectroscopy (ATR-FTIR, Thermo Nicolet iS50, Thermo Fisher Scientific, Waltham, MA, USA). Spectra were recorded over the wavenumber range of 400–4000 cm^−1^ with 64 accumulated scans.

#### 2.4.5. Ultraviolet Protection Properties

The ultraviolet (UV) protection properties of untreated and surface-modified cotton fabrics dyed with lignin, with and without silver incorporation, were evaluated using a UV–Vis spectrophotometer (M550SPF, Spectronic CamSpec Ltd., Leeds, West Yorkshire, UK) according to AATCC TM183-2010 [23]. UV transmittance in the UV-A and UV-B regions was measured over the wavelength range of 280–400 nm. The ultraviolet protection factor (*UPF*) values were calculated from the weighted average UV transmittance across this wavelength range using the standard AATCC equation. The reported *UPF* values represent the average of three independent measurements. The UV protection performance of the fabrics was classified according to the *UPF* rating categories defined in the AATCC standard.

#### 2.4.6. Evaluation of Antibacterial Properties of the Treated Fabrics

The antibacterial activity of the untreated and treated cotton fabrics was evaluated according to the AATCC TM100-2019 standard [24] using *Staphylococcus aureus* ATCC 6538 (*S. aureus*) as the test microorganism. Fabric specimens (approximately 1.0 ± 0.1 g) were sterilized by autoclaving at 121.0 °C for 15 min prior to testing.

Each sample was inoculated with a known concentration of bacterial suspension prepared in nutrient broth (1:20 dilution) containing 0.05% Triton X-100 to ensure uniform distribution. The inoculated specimens were incubated at 37.0 ± 2.0 °C for 24 h in closed containers.

After the incubation period, bacteria were recovered from the fabric samples using Dey–Engley neutralizing broth, followed by serial dilution and plating on nutrient agar. The number of viable bacteria was determined by counting colony-forming units (CFU).

The percentage reduction in bacteria was calculated using untreated cotton fabric as the control according to the following equation:

$$\mathrm{Bacterial}\;\mathrm{reduction}\;(\%)=100\times \frac{B-A}{B}$$

where *B* is the number of bacteria recovered from the untreated control specimen after 24 h of incubation, and *A* is the number of bacteria recovered from the treated specimen under the same conditions.

#### 2.4.7. Wash Durability

The antibacterial durability of the treated cotton fabrics was evaluated after a single washing cycle. The washing procedure was conducted according to ISO 105-C10:2006 (Test No. A) [25]. Fabric specimens (4 × 10 cm^2^) were washed in a 5.0 g/L standard detergent solution at 40.0 °C for 30 min using a liquor ratio of 1:50. After washing, the samples were rinsed thoroughly and air-dried at room temperature.

The antibacterial activity of the washed fabrics was then assessed against *Staphylococcus aureus* following the AATCC 100 test method [24]. The bacterial reduction was compared with that of unwashed samples to determine the retention of antibacterial performance after washing.

#### 2.4.8. Color Parameters and Color Strength

The color properties of cotton fabrics subjected to surface modification and dyed with lignin, with and without silver, were evaluated using a spectrophotometer under D65 standard illuminant conditions. Color parameters were determined based on the CIE L*a*b* color space, where L* represents lightness, a* corresponds to the red–green axis, and b* corresponds to the yellow–blue axis. At least three measurements at different positions on each sample were conducted. Color strength (K/S) values were calculated from the reflectance data using the Kubelka–Munk equation [26]:

$$\frac{K}{S}=\frac{{\left(1-R\right)}^{2}}{2R}$$

where *R* is the reflectance of the sample, and *K* and *S* represent the absorption and scattering coefficients, respectively. The maximum *K*/*S* value within the visible wavelength range of 400–700 nm was selected to represent the color depth of each sample.

#### 2.4.9. Tensile Strength

The tensile properties of the fabric samples were evaluated using a tensile strength tester (Zwick Roell, ZOO5TN, ZwickRoell GmbH & Co. KG, Ulm, Germany) in accordance with ASTM D5035-11 (2019) [27]. Fabric specimens were cut to dimensions of 5 × 15 cm^2^. Tensile testing was conducted using a gauge length of 7.5 cm, a crosshead speed of 300 mm/min, and a 5.0 kN load cell. The reported tensile strength values represent the average of ten specimens tested in each direction (warp and weft).

#### 2.4.10. Statistical Analysis

Statistical analysis was performed using one-way analysis of variance (ANOVA) followed by Tukey’s honestly significant difference (HSD) post hoc test using Origin 2025b (OriginLab Corporation, Northampton, MA, USA). Differences were considered statistically significant at *p* < 0.05. All results are presented as mean ± standard deviation. Most experiments were conducted in triplicate (*n* = 3), while tensile strength measurements were performed using ten independent specimens (*n* = 10). The statistical analysis was performed under the standard assumptions of ANOVA.

## 3. Results

### 3.1. Surface Morphology and Elemental Analysis of Treated Cotton Fabrics

**Figure 2:**
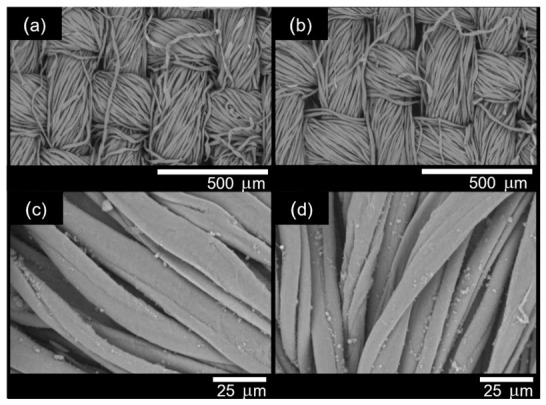
SEM images of Cot-αCS-L-Ag and Cot-βCS-L-Ag at magnifications of 100× (**a**,**b**) and 1000× (**c**,**d**).

The surface morphology and elemental distribution of cotton fabrics subjected to surface modification and subsequently dyed with silver-containing lignin were investigated using SEM coupled with EDS, as shown in Figure 2. At a low magnification of 100× (Figure 2a,b), both treated fabrics exhibited a woven structure characteristic of cotton textiles, in which the yarns appeared twisted, with some straightened fiber segments and occasional fiber protrusion from the yarn bundles [28]. These features are typical of cotton fabrics after chemical surface modification and dyeing processes. No significant structural damage or collapse of the fabric structure was observed, indicating that the surface treatments did not adversely affect the macroscopic integrity of the cotton fibers. At higher magnification 1000× (Figure 2c,d), discrete particulate features were clearly observed on the fiber surfaces of both samples. These particles are likely associated with deposited lignin-mediated silver-containing species formed during the dyeing process.

**Figure 3:**
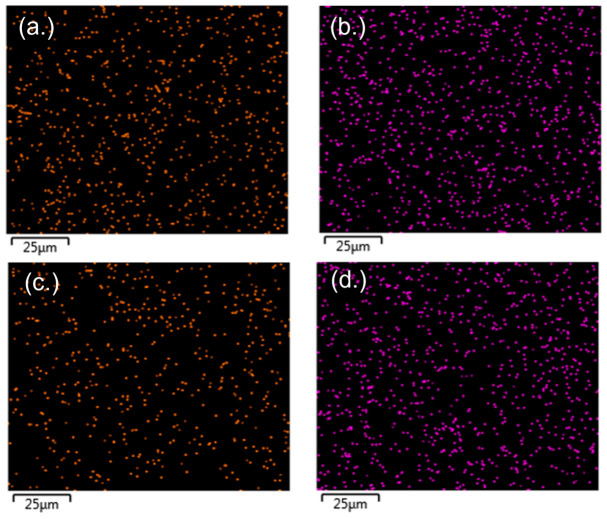
SEM–EDS elemental mapping images of nitrogen (N) and silver (Ag) distribution on treated cotton fabrics: (**a**) N-map and (**b**) Ag-map of Cot-αCS-L-Ag; (**c**) N-map and (**d**) Ag-map of Cot-βCS-L-Ag.

EDS elemental mapping further confirmed the presence and distribution of nitrogen and silver species on the treated cotton fabrics (Figure 3). Nitrogen signals were observed on both treated fabrics, providing supportive evidence consistent with the presence of chitosan-based coating layers on the cotton surface. Ag mapping signals were observed in both α- and β-chitosan-modified fabrics.

To obtain a more accurate quantification of silver loading, microwave digestion followed by ICP–OES analysis was performed. The results revealed that the silver content of Cot-αCS-L-Ag was 40.7 ± 0.3 mg/kg, while that of Cot-βCS-L-Ag was 14.7 ± 0.2 mg/kg. These findings confirm that both systems contained silver, although the β-chitosan-modified fabric exhibited substantially lower silver loading.

### 3.2. Morphological Properties of Lignin-AgNPs

**Figure 4:**
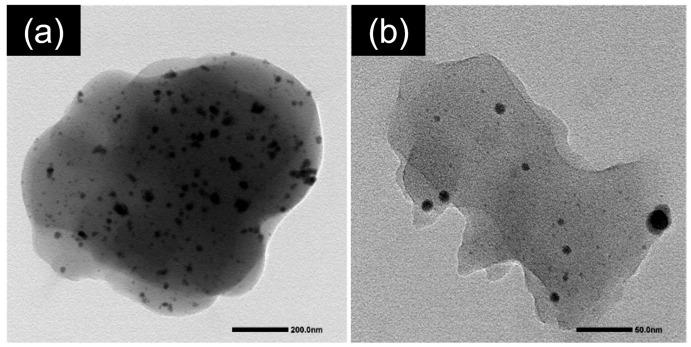
TEM images of particles extracted by ultrasonication from cotton fabrics surface-modified with (**a**) Cot-αCS-L-Ag and (**b**) Cot-βCS-L-Ag.

Figure 4 presents the TEM images of AgNPs extracted from cotton fabrics modified with α- and β-chitosan and subsequently dyed with lignin, which may act as a reducing agent for silver ions.

The TEM images revealed distinct differences in nanoparticle characteristics between the two systems. The nanoparticles obtained from the α-chitosan-modified fabric exhibited a larger average diameter (13.55 ± 7.31 nm, *n* = 237) and a relatively high observable particle density, with aggregation observed in some regions. In contrast, the β-chitosan-modified fabric showed smaller measurable nanoparticles (4.25 ± 3.57 nm, *n* = 30), with fewer detectable particles and localized aggregation in certain areas. The limited number of measurable nanoparticles observed in the β-chitosan sample was consistent with the lower silver loading determined by ICP-OES. Similar aggregation behavior of lignin-mediated AgNP systems has also been reported in previous lignin/cellulose composite fibers, where partial clustering of AgNPs on the fiber surface was observed after in situ reduction. This phenomenon suggests that lignin-mediated AgNP formation may lead to heterogeneous nanoparticle deposition rather than perfectly uniform dispersion, particularly in polymeric coating systems containing aromatic lignin domains [13].

These observations suggest that α-chitosan may provide a more compact and structurally stable matrix for AgNP retention. Previous studies have reported that α-chitosan generally exhibits higher crystallinity and stronger intermolecular hydrogen bonding than β-chitosan due to its antiparallel chain packing arrangement [19,20]. This structural difference may contribute to the greater number of observable nanoparticles remaining associated with the extracted fragments after ultrasonication. In contrast, β-chitosan, which possesses a more open and less crystalline structure, was associated with smaller measurable nanoparticles and lower observable nanoparticle density.

### 3.3. FTIR Spectra of Untreated and Chitosan-Treated Cotton Fabrics

**Figure 5:**
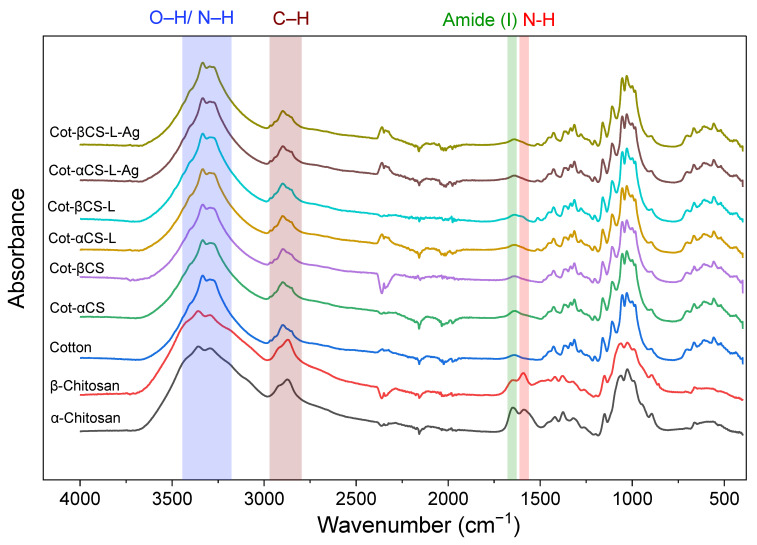
ATR–FTIR spectra of α-chitosan, β-chitosan, untreated cotton, chitosan-modified cotton fabrics (Cot-αCS and Cot-βCS), lignin-treated cotton fabrics (Cot-αCS-L and Cot-βCS-L), and lignin–Ag-treated cotton fabrics (Cot-αCS-L-Ag and Cot-βCS-L-Ag).

Figure 5 presents the ATR–FTIR spectra of untreated cotton, α-chitosan (αCS), β-chitosan (βCS), chitosan-treated cotton fabrics (Cot-αCS and Cot-βCS), and lignin-treated samples with and without silver incorporation. A broad absorption band centered around ~3400 cm^−1^, attributed to overlapping O–H and N–H stretching vibrations [29], was observed in all samples. This band is characteristic of polysaccharide-based materials and is associated with hydrogen bonding among hydroxyl groups in cellulose and amino/hydroxyl groups in chitosan. Similar spectral features were observed among the treated fabrics, indicating substantial overlap of functional groups from cellulose, chitosan, and lignin components.

In addition, absorption bands in the range of 2850–2950 cm^−1^, corresponding to C–H stretching vibrations of aliphatic –CH and –CH_2_ groups [29], were present in all samples. Bands around ~1650 cm^−1^ and ~1590 cm^−1^, typically assigned to amide I and N–H bending/C–N stretching vibrations of chitosan [30], were also detected in the chitosan-containing samples. Lignin-containing fabrics exhibited generally similar spectral profiles, although slight variations in band intensity and broadening were observed after lignin treatment. No distinct new absorption bands specifically attributable to silver incorporation were clearly identified, likely due to the relatively low silver loading and the overlapping nature of the ATR–FTIR spectra. However, no consistent peak shifts or sufficiently distinct changes could be quantitatively resolved. This limitation is likely due to the substantial overlap of absorption bands among cellulose, chitosan, and lignin components. Therefore, ATR–FTIR analysis in this study was primarily used as supportive evidence for surface modification rather than definitive proof of specific intermolecular interactions.

### 3.4. Ultraviolet Protection Efficiency and Coating Add-On

The *UPF* values of untreated and treated cotton fabrics are presented in Table 1. Untreated cotton exhibited relatively low UV protection due to the porous woven structure and the limited intrinsic UV absorption ability of cellulose fibers [31]. After surface modification with chitosan and subsequent lignin treatment, the UV protection properties of the fabrics improved significantly.

Among the treated samples, Cot-αCS-L-Ag exhibited higher *UPF* values than Cot-βCS-L-Ag. This behavior was consistent with the higher solid add-on and silver content observed in the α-chitosan-treated fabrics. The denser coating structure associated with α-chitosan may contribute to enhanced UV shielding by increasing both UV absorption and light scattering at the fiber surface. In contrast, the β-chitosan system, which possesses a more open and less ordered structure, may form a less compact coating layer, resulting in comparatively lower UV protection performance.

The improved UV protection after lignin incorporation can also be attributed to the intrinsic UV-absorbing characteristics of lignin. Lignin contains aromatic rings, conjugated structures, and phenolic functional groups capable of absorbing ultraviolet radiation, particularly within the UV-B region [6]. Previous studies have similarly reported that lignin-based textile coatings can effectively enhance UV shielding performance. For example, bamboo ethanol lignin-coated cotton fabrics exhibited a *UPF* value of 48.2 together with UVA and UVB blocking efficiencies approaching 100%, demonstrating the strong UV-absorbing capability of lignin-derived aromatic structures. In addition, lignin deposition on fiber surfaces may increase optical scattering and reduce direct UV penetration through the fabric structure [32].

### 3.5. Antibacterial Activity of Untreated and Modified Cotton Fabrics

The antibacterial activity of untreated and modified cotton fabrics against *S. aureus* is summarized in Table 2. Both Cot-αCS and Cot-βCS exhibited bacterial reduction values of >99.99%, confirming the strong intrinsic antibacterial activity of chitosan-coated fabrics. This behavior is commonly attributed to protonated amino groups (–NH_3_^+^) on chitosan, which can interact with negatively charged bacterial cell membranes and disrupt cellular functions [33]. Previous studies have similarly reported high antibacterial efficiency of chitosan-treated cotton fabrics against *S. aureus* [29,34].

After subsequent lignin and silver treatment, the antibacterial activity decreased to 68.69 ± 11.77% for Cot-αCS-L-Ag and 82.62 ± 7.46% for Cot-βCS-L-Ag. Although both lignin and AgNPs have been reported to exhibit antibacterial activity [35,36], the present results suggest that antibacterial performance in these systems depends more strongly on the accessibility of active sites than on the total amount of active components. The incorporation of lignin and silver species may partially shield protonated amino groups and reduce their direct interaction with bacterial cells.

A significant difference was observed between the α- and β-chitosan systems. Despite its lower silver content determined by ICP–OES analysis, Cot-βCS-L-Ag exhibited significantly higher antibacterial activity than Cot-αCS-L-Ag. Previous studies have reported that β-chitosan possesses a less ordered and less crystalline structure than α-chitosan, resulting in greater structural accessibility and reactivity [20]. In addition, the antibacterial performance of chitosan-treated cotton fabrics has been reported to depend not only on the amount of deposited chitosan but also on the structural characteristics of the coating layer [37]. The more open structure of β-chitosan may therefore promote better surface accessibility and distribution of active components, consistent with the smaller nanoparticle size and lower observable aggregation shown in Figure 4.

After one washing cycle, the antibacterial reduction in Cot-αCS-L-Ag decreased to 41.87%, whereas Cot-βCS-L-Ag maintained high antibacterial activity (99.36%). This behavior may be associated with differences in coating structure and accessibility. Partial removal or rearrangement of loosely bound lignin and silver species during washing may increase the exposure of protonated amino groups in the β-chitosan system, while the denser α-chitosan coating structure may restrict such exposure.

Overall, the results suggest that antibacterial performance in these multifunctional coating systems is governed more strongly by the accessibility and distribution of active sites than by the total silver content alone.

### 3.6. Colorimetric Analysis and Color Strength of Dyed Fabrics

The colorimetric parameters of cotton fabrics dyed with lignin-based dyes at pH 4.0 are summarized in Table 3. The lignin-dyed cotton (Cot-L) exhibited a high L* value (89.20) and a very low *K*/*S* value (0.14), indicating limited dye uptake. This can be attributed to the weak interaction between lignin and the cotton surface. At pH 4.0, carboxylic groups in lignin (pKa ≈ 3–5) are partially deprotonated, imparting a negative charge to the lignin molecules [22]. Meanwhile, cotton fibers, with an isoelectric point of approximately pH 2.9 [38], also carries a net negative charge under these conditions. The resulting electrostatic repulsion between negatively charged lignin and cotton surfaces leads to low affinity and poor dye uptake [39].

Chitosan pretreatment significantly enhanced color depth, as reflected by lower L* and higher *K/S* values in both αCS-L and βCS-L samples. This improvement is attributed to protonated amino groups (–NH_3_^+^) in chitosan, which promote electrostatic interactions with lignin. Such enhancement in dyeability of cotton after chitosan modification has been widely reported for natural dye systems, where chitosan introduces cationic sites that improve dye–fiber affinity [40,41]. Notably, Cot-βCS-L exhibited slightly higher *K/S* values despite its lower coating add-on, suggesting that dye uptake is governed not only by coating amount but also by structural accessibility. The less ordered structure of β-chitosan likely facilitates lignin diffusion and binding, whereas the more compact α-chitosan structure may restrict accessibility.

The incorporation of silver slightly decreased *K*/*S,* likely due to partial occupation of binding sites. AgNPs may contribute marginally to color through their characteristic yellowish appearance; however, lignin remains the dominant contributor.

These results are consistent with the add-on and UV protection data, where the higher add-on in the α-chitosan system leads to a denser coating and improved UV shielding, but reduced accessibility of functional sites. In contrast, the more open structure of the β-chitosan system enhances both dye uptake and antibacterial performance, despite its lower add-on and silver content.

Overall, the results demonstrate that functional performance is governed primarily by the distribution and accessibility of active components rather than their total loading.

### 3.7. Mechanical Performance

The tensile properties of untreated and treated cotton fabrics are presented in Table 4. In the warp direction, the tensile strength of Cot-αCS-L-Ag (423 ± 19 N) was significantly higher than that of untreated cotton (398 ± 28 N) and Cot-βCS-L-Ag (398 ± 30 N) (*p* < 0.05), while no significant difference was observed between the latter two samples. This indicates that the α-chitosan system contributes to enhanced load-bearing capacity, likely due to its higher coating add-on and the formation of a more compact and reinforcing layer on the fiber surface.

In contrast, no significant differences in elongation at break were observed among the samples in the warp direction (*p* > 0.05), suggesting that the coating does not adversely affect the extensibility of the load-bearing yarns.

In the weft direction, a different trend was observed. The tensile strength of Cot-βCS-L-Ag (242 ± 11 N) was significantly higher than that of both untreated cotton (220 ± 22 N) and Cot-αCS-L-Ag (215 ± 14 N), while no significant difference was found between cotton and Cot-αCS-L-Ag. This behavior may be attributed to differences in coating structure. The more open and less ordered β-chitosan structure may facilitate improved stress distribution and load transfer, whereas the relatively compact α-chitosan layer primarily contributes to reinforcement rather than enhancing stress redistribution under transverse loading. This directional dependence reflects the intrinsic structural differences between warp and weft yarns, where warp yarns act as the primary load-bearing elements, while weft yarns undergo greater deformation and benefit more from a flexible and compliant coating.

A clear distinction was also observed in elongation at break in the weft direction. Cot-βCS-L-Ag exhibited the highest elongation (32.6 ± 1.5%), followed by Cot-αCS-L-Ag (29.1 ± 1.6%) and untreated cotton (27.7 ± 1.7%), with all differences being statistically significant (*p* < 0.05). This result indicates that the β-chitosan system enhances fabric flexibility more effectively, likely due to its less ordered structure, which allows greater chain mobility. In contrast, the α-chitosan coating also improves elongation compared to untreated cotton, suggesting that the polymeric coating imparts a degree of flexibility while maintaining its reinforcing effect.

Overall, the results demonstrate a clear relationship among coating add-on, silver content, nanoparticle characteristics, and functional performance. The α-chitosan system, which exhibited a higher coating add-on, resulted in greater silver retention and the formation of larger nanoparticles. This denser and more compact coating structure enhanced UV protection but may limit the surface accessibility of active components. As a result, antibacterial performance and color strength were not fully realized despite the higher silver content.

In contrast, the β-chitosan system, characterized by a lower coating add-on and more open structure, promoted the formation of smaller measurable nanoparticles. Although the total silver content was lower, the more open coating structure may promote greater surface accessibility and distribution of active components, which was consistent with the enhanced antibacterial activity and higher color strength observed in this system. These findings suggest that functional performance is influenced not only by the quantity of deposited materials but also by their distribution and potential accessibility within the coating layer. Therefore, the structural differences between α- and β-chitosan play an important role in controlling the structure–property relationships of lignin–silver-modified cotton fabrics.

## 4. Conclusions

This study investigated the influence of chitosan polymorphs on multifunctional cotton fabrics dyed with lignin in the presence of silver ions. Chitosan was introduced to improve adhesion to cotton and enhance lignin uptake, while lignin simultaneously functioned as a reducing agent for the in situ formation of silver nanoparticles (AgNPs).

The results demonstrated that chitosan polymorphism significantly affected nanoparticle characteristics and textile performance. The more compact α-chitosan structure was associated with higher silver loading and larger measurable nanoparticles, whereas β-chitosan produced smaller measurable nanoparticles with lower silver loading. Despite the lower silver content, the β-chitosan system exhibited superior antibacterial activity against *S. aureus*, suggesting that antibacterial performance may depend not only on total silver loading, but also on nanoparticle distribution and the accessibility of active components within the coating layer. In contrast, the denser α-chitosan coating structure provided superior UV protection. The coating treatments also influenced the tensile behavior of the fabrics in a direction-dependent manner.

Overall, this study provides new insight into the role of chitosan polymorphism in lignin-mediated AgNP textile systems and highlights the importance of coating structure in determining multifunctional performance. These findings contribute to the development of sustainable lignin-based textile finishing approaches for antibacterial and UV-protective applications. From a practical perspective, the α-chitosan system may be more suitable for UV-protective textiles due to its denser coating structure and higher UV shielding performance, whereas the β-chitosan system may be more favorable for antibacterial textiles because of its enhanced antibacterial activity.

However, several limitations should be acknowledged. Nanoparticle accessibility and surface interactions were inferred from indirect evidence rather than direct surface characterization techniques. In addition, the antibacterial evaluation was limited to *S. aureus* and short-term washing durability. Future studies should therefore investigate broader antimicrobial performance, long-term durability, and advanced surface characterization to further clarify nanoparticle behavior in lignin–silver–chitosan systems.

## Figures and Tables

**Table 1:** Ultraviolet protection factor (*UPF*) of treated cotton fabrics measured according to AATCC TM183-2010, along with solid add-on (%) (mean ± standard deviation, *n* = 3).

Sample	*UPF*	Solid Add-On (%)
Untreated cotton	6.7 ± 1.5 ^c^	–
Cot-αCS-L-Ag	25.5 ± 3.2 ^a^	7.95 ± 1.95 ^a^
Cot-βCS-L-Ag	20.5 ± 3.8 ^b^	2.07 ± 1.46 ^b^

Different superscript letters within the same column indicate significant differences (*p* < 0.05, Tukey’s HSD test).

**Table 2:** Antibacterial activity against *S. aureus* evaluated according to AATCC TM100-2019, expressed as bacterial reduction (%) before and after washing (mean ± standard deviation, *n* = 3).

Sample	Bacterial Reduction (%)	
	Before Washing	After Washing
Untreated cotton	0	–
Cot-αCS	>99.99	–
Cot-βCS	>99.99	–
Cot-αCS-L-Ag	68.69 ± 11.77 ^b^	41.87 ± 16.52 ^b^
Cot-βCS-L-Ag	82.62 ± 7.46 ^a^	99.36 ± 1.03 ^a^

Different superscript letters within each column indicate significant differences (*p* < 0.05, Tukey’s HSD test). “–” indicates that the test was not performed.

**Table 3:** Colorimetric parameters (L*, a*, b*) and color strength (*K*/*S*) of untreated and treated cotton fabrics with corresponding fabric images (mean ± standard deviation, *n* = 3).

Sample	Color Parameter				Fabric Photo
	L*	a*	b*	*K*/*S*	
Cot-L	89.20	1.08	4.50	0.14 ± 0 ^d^	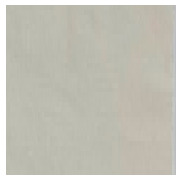
Cot-αCS-L	68.19	6.17	18.00	1.70 ± 0.04 ^b^	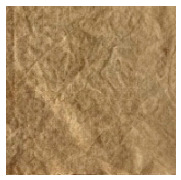
Cot-αCS-L-Ag	67.95	4.13	15.91	1.55 ± 0.03 ^c^	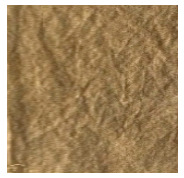
Cot-βCS-L	68.01	6.21	18.81	1.79 ± 0.02 ^a^	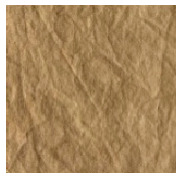
Cot-βCS-L-Ag	66.01	4.38	15.81	1.72 ± 0.04 ^b^	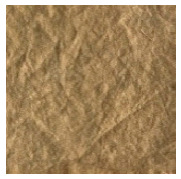

Different superscript letters indicate significant differences (*p* < 0.05, Tukey’s HSD test).

**Table 4:** Tensile properties (force and elongation) of untreated and treated cotton fabrics measured according to ASTM D5035-11 (2019) (mean ± standard deviation, *n* = 10).

Sample	Warp		Weft	
	Force (N)	Elongation (%)	Force (N)	Elongation (%)
Cotton	398 ± 28 ^b^	12.0 ± 0.9 ^a^	220 ± 22 ^b^	27.7 ± 1.7 ^c^
Cot-αCS-L-Ag	423 ± 19 ^a^	12.4 ± 0.5 ^a^	215 ± 14 ^b^	29.1 ± 1.6 ^b^
Cot-βCS-L-Ag	398 ± 30 ^b^	11.8 ± 0.6 ^a^	242 ± 11 ^a^	32.6 ± 1.5 ^a^

Different superscript letters within each column indicate significant differences (*p* < 0.05, Tukey’s HSD test).

## Data Availability

The raw data supporting the conclusions of this article will be made available by the authors on request.

## References

[1] Zhang Z., Zhao C., Tang X., Yan A., Luo Y., Yang F., Wang Y. (2024). Synthesis and identification of Chitosan@ lignin hydrogel and its effect on seed germination. Eur. Phys. J. Plus.

[2] Neiva D.M., Rencoret J., Marques G., Gutiérrez A., Gominho J., Pereira H., Del Río J.C. (2020). Lignin from tree barks: Chemical structure and valorization. ChemSusChem.

[3] Katahira R., Elder T., Beckham G. (2018). Chapter 1: A brief introduction to lignin structure. Lignin Valorization: Emerging Approaches.

[4] Ali M.A.-S., Abdel-Moein N.M., Owis A.S., Ahmed S.E., Hanafy E.A. (2024). Eco-friendly lignin nanoparticles as antioxidant and antimicrobial material for enhanced textile production. Sci. Rep..

[5] Li Y., Yang D., Li P., Li Z. (2022). Lignin as a multi-functional agent for the synthesis of Ag nanoparticles and its application in antibacterial coatings. J. Mater. Res. Technol..

[6] Sadeghifar H., Ragauskas A. (2020). Lignin as a UV light blocker—A review. Polymers.

[7] Solihat N.N., Hidayat A.F., Taib M.N.A.M., Hussin M.H., Lee S.H., Ghani M.A.A., Edrus S.S.O.A., Vahabi H., Fatriasari W. (2022). Recent developments in flame-retardant lignin-based biocomposite: Manufacturing, and characterization. J. Polym. Environ..

[8] Ito N.M., de Andrade Mendes Filho A., dos Santos D.J., dos Santos L.T. (2024). Synthesis of silver nanoparticles using modified lignin as a reducing agent. Next Mater..

[9] Shankar S., Rhim J.-W., Won K. (2018). Preparation of poly (lactide)/lignin/silver nanoparticles composite films with UV light barrier and antibacterial properties. Int. J. Biol. Macromol..

[10] Aadil K.R., Pandey N., Mussatto S.I., Jha H. (2019). Green synthesis of silver nanoparticles using acacia lignin, their cytotoxicity, catalytic, metal ion sensing capability and antibacterial activity. J. Environ. Chem. Eng..

[11] Jaiswal L., Shankar S., Rhim J.-W., Hahm D.-H. (2020). Lignin-mediated green synthesis of AgNPs in carrageenan matrix for wound dressing applications. Int. J. Biol. Macromol..

[12] Kędziora A., Speruda M., Krzyżewska E., Rybka J., Łukowiak A., Bugla-Płoskońska G. (2018). Similarities and differences between silver ions and silver in nanoforms as antibacterial agents. Int. J. Mol. Sci..

[13] Chen Y., Wang S., Hu G., Kong F., Hu J. (2025). Ultraviolet protection and antibacterial properties of textile fabric made of silver nanoparticles/alkaline lignin/regenerated cellulose fiber. Ind. Crops Prod..

[14] Zhou B.C.-E., Kan C.-w., Sun C., Du J., Xu C. (2019). A review of chitosan textile applications. AATCC J. Res..

[15] Panda P.K., Dash P., Yang J.-M., Chang Y.-H. (2022). Development of chitosan, graphene oxide, and cerium oxide composite blended films: Structural, physical, and functional properties. Cellulose.

[16] Claesson P.M., Ninham B.W. (1992). pH-dependent interactions between adsorbed chitosan layers. Langmuir.

[17] Mahbub M.A., Mahmud M.H., Ahona M.J., Ahmed T., Ashraf S.M., Sultana J.A., Hasan M., Islam M.T. (2024). Chitosan as a cationizing agent in pigment dyeing of cotton fabric. Carbohydr. Polym. Technol. Appl..

[18] García-González A., Zavala-Arce R.E., Avila-Pérez P., Salazar-Rábago J.J., Garcia-Rivas J.L., Barrera-Díaz C.E. (2025). Effect of Protonated Media on Dye Diffusion in Chitosan–Cellulose-Based Cryogel Beads. Gels.

[19] Barikani M., Oliaei E., Seddiqi H., Honarkar H. (2014). Preparation and application of chitin and its derivatives: A review. Iran. Polym. J..

[20] Jung J., Zhao Y. (2013). Impact of the structural differences between α-and β-chitosan on their depolymerizing reaction and antibacterial activity. J. Agric. Food Chem..

[21] Marković D., Petkovska J., Mladenovic N., Radoičić M., Rodriguez-Melendez D., Ilic-Tomic T., Radetić M., Grunlan J.C., Jordanov I. (2023). Antimicrobial and UV protective chitosan/lignin multilayer nanocoating with immobilized silver nanoparticles. J. Appl. Polym. Sci..

[22] Ravishankar K., Venkatesan M., Desingh R.P., Mahalingam A., Sadhasivam B., Subramaniyam R., Dhamodharan R. (2019). Biocompatible hydrogels of chitosan-alkali lignin for potential wound healing applications. Mater. Sci. Eng. C.

[23] (2010). Transmittance or Blocking of Erythemally Weighted Ultraviolet Radiation Through Fabrics.

[24] (2019). Assessment of Antibacterial Finishes on Textile Materials.

[25] (2006). Textiles—Tests for Colour Fastness—Part C10: Colour Fastness to Washing with Soap or Soap and Soda (Test No. A).

[26] Kubelka P. (1931). Ein beitrag zur optik der farbanstriche. Z. Tech. Phys..

[27] (2019). Standard Test Method for Breaking Force and Elongation of Textile Fabrics (Strip Method).

[28] Zhu J., Li H., Wang Y., Wang Y., Yan J. (2021). Preparation of Ag NPs and its multifunctional finishing for cotton fabric. Polymers.

[29] Rahman Bhuiyan M., Hossain M.A., Zakaria M., Islam M., Zulhash Uddin M. (2017). Chitosan coated cotton fiber: Physical and antimicrobial properties for apparel use. J. Polym. Environ..

[30] Wen M., Tao R., Jiang J., Chen Y., Wang Y. (2025). Characterization and anti-Vibrio activity of citral-chitosan Schiff base complexed tannic acid-zinc slow-release bacteriostatic formulations. LWT.

[31] Kibria G., Repon M.R., Hossain M.F., Islam T., Jalil M.A., Aljabri M.D., Rahman M.M. (2022). UV-blocking cotton fabric design for comfortable summer wears: Factors, durability and nanomaterials. Cellulose.

[32] Dong X., Gao L., Xiao T., Zhang J., Ping Q. (2025). Green and sustainable fabrication of UV-resistance and antioxidant cotton via self-crosslinking bamboo ethanol lignin. Ind. Crops Prod..

[33] Li J., Zhuang S. (2020). Antibacterial activity of chitosan and its derivatives and their interaction mechanism with bacteria: Current state and perspectives. Eur. Polym. J..

[34] Öktem T. (2003). Surface treatment of cotton fabrics with chitosan. Color. Technol..

[35] Das A.K., Mitra K., Conte A.J., Sarker A., Chowdhury A., Ragauskas A.J. (2024). Lignin-A green material for antibacterial application—A review. Int. J. Biol. Macromol..

[36] Tang S., Zheng J. (2018). Antibacterial activity of silver nanoparticles: Structural effects. Adv. Healthc. Mater..

[37] Shin Y., Yoo D., Jang J. (2001). Molecular weight effect on antimicrobial activity of chitosan treated cotton fabrics. J. Appl. Polym. Sci..

[38] Grancaric A.M., Tarbuk A., Pusic T. (2005). Electrokinetic properties of textile fabrics. Color. Technol..

[39] Lehtonen J., Johansson P., Babaeipour S., Nousiainen P., Periyasamy A.P., Österberg M., Vuorinen T. (2025). Application of kraft lignin as a colorant for screen printing on cotton. Ind. Crops Prod..

[40] Mansour R., Ben Ali H. (2021). Investigating the use of chitosan: Toward improving the dyeability of cotton fabrics dyed with Roselle (*Hibiscus sabdariffa* L.). J. Nat. Fibers.

[41] Ke G., Zhu K., Chowdhury M.H. (2021). Dyeing of cochineal natural dye on cotton fabrics treated with oxidant and chitosan. J. Nat. Fibers.

